# Supplementary material for: Genetic and morphometric divergence in the Garnet-Throated Hummingbird Lamprolaima rhami (Aves: Trochilidae)
Source: PeerJ. 2018 Oct 19;6:e5733. doi: 10.7717/peerj.5733 (PMC6197039; doi:10.7717/peerj.5733)
Supplement: Table S1 — Localities, geographic groups (GG), coordinates and biological collections of Lamprolaima rhami tissue samples. [file peerj-06-5733-s001.pdf]

Supplemental information S1

Localities, geographic groups (GG), coordinates and biological collections of *Lamprolaima rhami* tissue samples.

GG	Collection number	State/Department	Locality	Latitude	Longitude	BC
SMO	AHC042	Puebla	Tetela	19.88685	-97.69383	MZFC
SMO	AHC057	Puebla	Tetela	19.88685	-97.69383	MZFC
SMO	AHC067	Puebla	Tetela	19.88685	-97.69383	MZFC
SMO	AHC073	Puebla	Tetela	19.88685	-97.69383	MZFC
SMO	AHC077	Puebla	Tetela	19.88685	-97.69383	MZFC
SMO	CONACYT750	Oaxaca	Puerto de La Soledad	18.165	-96.99667	MZFC
SMO	CONACYT752	Oaxaca	Puerto de La Soledad	18.165	-96.99667	MZFC
SMO	CONACYT756	Oaxaca	Puerto de La Soledad	18.165	-96.99667	MZFC
SMO	CONACYT762	Oaxaca	Puerto de La Soledad	18.165	-96.99667	MZFC
SMO	CONACYT777	Oaxaca	Puerto de La Soledad	18.165	-96.99667	MZFC
SMO	CONACYT781	Oaxaca	Puerto de La Soledad	18.165	-96.99667	MZFC
SMO	OMVP006	Oaxaca	Puerto de La Soledad	18.165	-96.99667	MZFC
SMO	OMVP013	Oaxaca	Puerto de La Soledad	18.165	-96.99667	MZFC
SMO	OMVP052	Oaxaca	Puerto de La Soledad	18.165	-96.99667	MZFC
SMO	MT374	Oaxaca	La Esperanza	17.515	-96.505	MZFC
SMO	B22001	Oaxaca	Distrito de Cuicatlán	17.856	-96.6456	MNS
SMO	OMVP919	Oaxaca	Distrito de Cuicatlán	17.846667	-96.75667	MZFC
SMO	OMVP959	Oaxaca	Distrito de Cuicatlán	17.846667	-96.75667	MZFC
SMO	OMVP1036	Oaxaca	San Martín Caballero	18.111667	-96.64	MZFC
SMO	OMVP1067	Oaxaca	San Martín Caballero	18.111667	-96.64	MZFC
SMO	OMVP1081	Oaxaca	San Martín Caballero	18.111667	-96.64	MZFC
SMO	OMVP1113	Oaxaca	San Martín Caballero	18.111667	-96.64	MZFC
MIA	MOL15-025	Oaxaca	Sierra de Miahuatlán	16.08972	-96.48548	MZFC
MIA	MOL15-027	Oaxaca	Sierra de Miahuatlán	16.08972	-96.48548	MZFC
GRO	AMT049	Guerrero	Carrizal de Bravo	17.676111	-99.88194	MZFC
GRO	AMT133	Guerrero	Carrizal de Bravo	17.676111	-99.88194	MZFC
GRO	AMT155	Guerrero	Carrizal de Bravo	17.676111	-99.88194	MZFC

GRO	ATO117	Guerrero	Carrizal de Bravo	17.676111	-99.88194	MZFC
GRO	ATO120	Guerrero	Carrizal de Bravo	17.676111	-99.88194	MZFC
GRO	MOLGRO164	Guerrero	Carrizal de Bravo	17.58668	-99.83707	MZFC
GRO	MOLGRO177	Guerrero	Carrizal de Bravo	17.58668	-99.83707	MZFC
GRO	MOLGRO195	Guerrero	Carrizal de Bravo	17.58668	-99.83707	MZFC
GRO	MOLGRO231	Guerrero	Carrizal de Bravo	17.58668	-99.83707	MZFC
CHIS	MOL13-052	Chiapas	Cerro Huitepec	16.738056	-92.68806	MZFC
CHIS	MOL13-076	Chiapas	Cerro Huitepec	16.738056	-92.68806	MZFC
CHIS	MOL13-119	Chiapas	Cerro Huitepec	16.738056	-92.68806	MZFC
CHIS	MOL13-132	Chiapas	Cerro Huitepec	16.738056	-92.68806	MZFC
CHIS	MOL13-297	Chiapas	Cerro Huitepec	16.738056	-92.68806	MZFC
CHIS	EAGT809	Chiapas	Cerro Mozotal	15.42	-92.34	MZFC
CHIS	EAGT813	Chiapas	Cerro Mozotal	15.42	-92.34	MZFC
CHIS	EAGT818	Chiapas	Cerro Mozotal	15.42	-92.34	MZFC
CHIS	ZRH397	Chiapas	Cerro Mozotal	15.42	-92.34	MZFC
CHIS	ZRH1008	Chiapas	Cerro Mozotal	15.42	-92.34	MZFC
CHIS	CRGA035	Chiapas	Cerro Boquerón	15.23	-92.30	MZFC
CHIS	EAGT828	Chiapas	Cerro Boquerón	15.23	-92.30	MZFC
CHIS	ZRH418	Chiapas	Cerro Boquerón	15.23	-92.30	MZFC
CHIS	ZRH419	Chiapas	Cerro Boquerón	15.23	-92.30	MZFC
CHIS	BMM875	Chiapas	Volcán Tacaná	15.06667	-92.08333	MZFC
CHIS	BMM877	Chiapas	Volcán Tacaná	15.06667	-92.08333	MZFC
CHIS	BONA90	Chiapas	Volcán Tacaná	15.06667	-92.08333	MZFC
CHIS	MVZ184738	El Progreso	Las Cabañas de Albores, Cerro Pinalón.	15.08385	-89.94305	MVZ
CHIS	RAJ101	Quetzaltenango	Zunil	14.748512	-91.48001	MVZ
CHIS	JF2859	El Quiche	Chimel, Uspatan.	15.46670	-90.7764	MVZ
CHIS	ZRH789	El Quiche	Chimel, Uspatan.	15.46670	-90.7764	MVZ

BC: Biological Collection.
MZFC: Museo de Zoología, Facultad de Ciencias (Alfonso L. Herrera), Universidad Nacional Autónoma de México.
MNS: Museum of Natural Science, Louisiana State University.
MVZ: Museum of Vertebrate Zoology, University of California Berkeley.
