# Supplementary material for: Genetic and morphometric divergence in the Garnet-Throated Hummingbird Lamprolaima rhami (Aves: Trochilidae)
Source: PeerJ. 2018 Oct 19;6:e5733. doi: 10.7717/peerj.5733 (PMC6197039; doi:10.7717/peerj.5733)
Supplement: Table S2 — Localities, geographic groups (GG), coordinates and biological collections of Lamprolaima rhami voucher specimens used for morphometric measurements. Map: vouchers from localities with data of geographic coordinates. [file peerj-06-5733-s002.pdf]

Supplemental information S2

Localities, geographic groups (GG), coordinates and biological collections of *Lamprolaima rhami* voucher specimens used for morphometric measurements. Map: vouchers from localities with data of geographic coordinates.

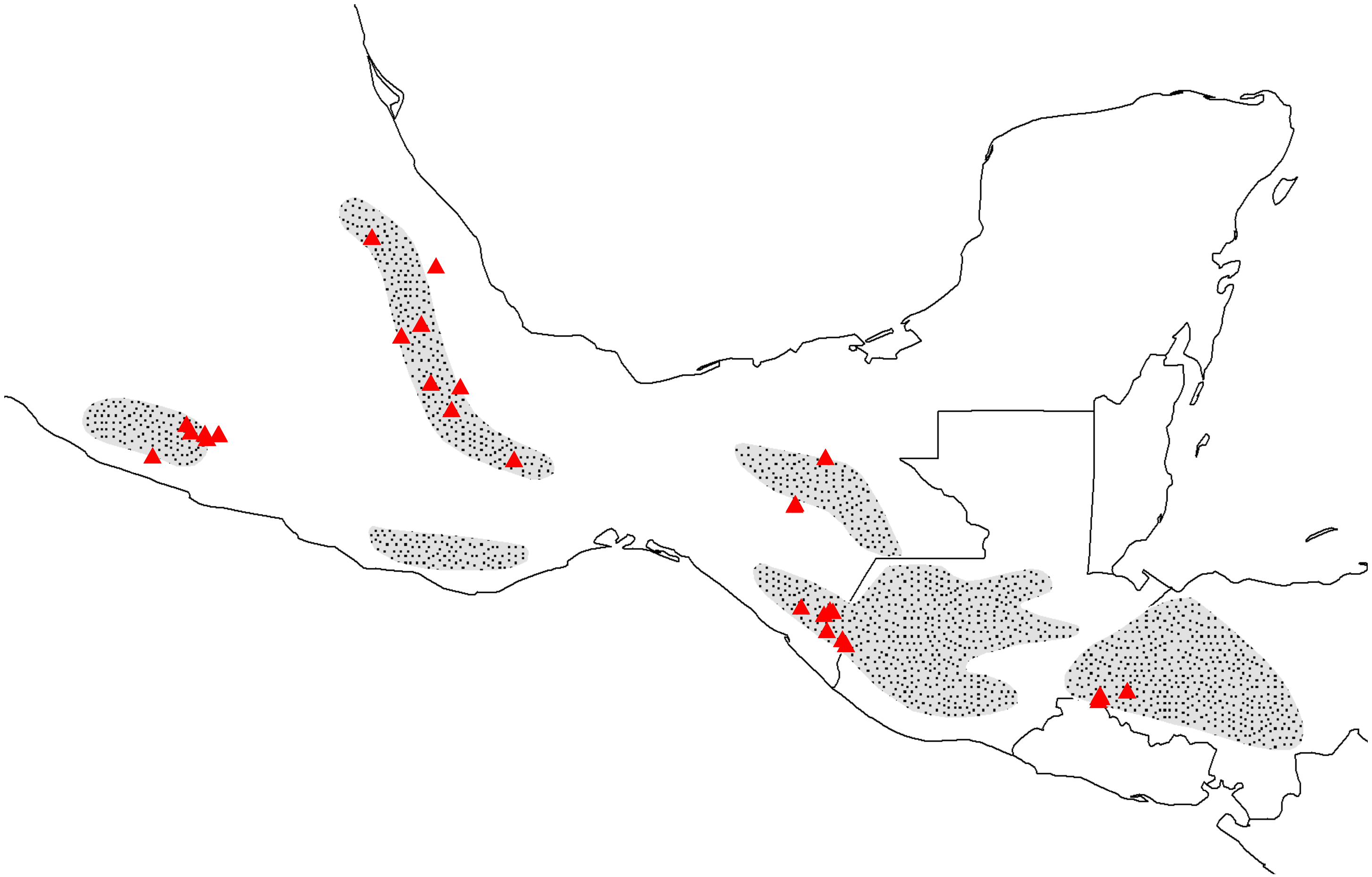

GG	ID	State/Department	Locality	Latitude	Longitude	BC	SEX
SMO	AHC067	Puebla	Tetela	19.88	-97.69	MZFC	M
SMO	AHC073	Puebla	Tetela	19.88	-97.69	MZFC	M
SMO	AHC077	Puebla	Tetela	19.88	-97.69	MZFC	M
SMO	AHC184	Puebla	Tetela	19.88	-97.69	MZFC	M
SMO	46308	Veracruz	Jalapa de Oca	ND	ND	AMNH	M
SMO	46309	Veracruz	Jalapa	ND	ND	AMNH	M
SMO	10308	Veracruz	Orizaba	18.851014	-97.100839	MLZ	M
SMO	33014	Veracruz	La Puerta, 31 mi SW Orizaba by road	18.714682	-97.338439	MLZ	M
SMO	33018	Veracruz	La Puerta, 31 mi SW Orizaba by road	18.714682	-97.338439	MLZ	M
SMO	33289	Veracruz	La Puerta, 31 mi SW Orizaba by road	18.714682	-97.338439	MLZ	M
SMO	33290	Veracruz	La Puerta, 31 mi SW Orizaba by road	18.714682	-97.338439	MLZ	M
SMO	32951	Veracruz	La Puerta, 31 mi SW Orizaba by road	18.714682	-97.338439	MLZ	M
SMO	12286	Veracruz	Vicinity of Coatepec	19.5456514	-96.9285929	MCZ	M
SMO	31864	Veracruz	Orizaba	18.8506857	-97.1002655	MCZ	M
SMO	31865	Veracruz	Orizaba	18.8506857	-97.1002655	MCZ	M
SMO	78508	Veracruz	Orizaba	18.8506857	-97.1002655	MCZ	M
SMO	AGNS663	Oaxaca	Macuiltianguis	-96.55	17.53166667	MZFC	M
SMO	CONACYT762	Oaxaca	Puerto de La Soledad	18.16	-96.99	MZFC	M
SMO	CONACYT781	Oaxaca	Puerto de La Soledad	18.16	-96.99	MZFC	M
SMO	OMVP1173	Oaxaca	San Martín Caballero	-96.64	18.11166667	MZFC	M
SMO	OMVP1067	Oaxaca	San Martín Caballero	18.11	-96.64	MZFC	M
SMO	OMVP959	Oaxaca	Distrito de Cuicatlán	17.84	-96.75	MZFC	M
SMO	37225	Oaxaca	Oaxaca	ND	ND	AMNH	M
SMO	37224	Oaxaca	Oaxaca	ND	ND	AMNH	M
SMO	37226	Oaxaca	Oaxaca	ND	ND	AMNH	M
SMO	793417	Oaxaca	22 mi. N. of San Juan del Estado	ND	ND	AMNH	M

SMO	30931	Oaxaca	Moctum	17.247789	-96.004919	MLZ	M
SMO	30934	Oaxaca	Moctum	17.247789	-96.004919	MLZ	M
SMO	30928	Oaxaca	Moctum	17.247789	-96.004919	MLZ	M
SMO	30932	Oaxaca	Moctum	17.247789	-96.004919	MLZ	M
SMO	37247	Oaxaca	Moctum	17.247789	-96.004919	MLZ	M
SMO	30933	Oaxaca	Moctum	17.247789	-96.004919	MLZ	M
SMO	AHC042	Puebla	Tetela	19.88	-97.69	MZFC	F
SMO	AHC057	Puebla	Tetela	19.88	-97.69	MZFC	F
SMO	AHC164	Puebla	Tetela	19.88	-97.69	MZFC	F
SMO	10198	Veracruz	Orizaba	18.851014	-97.100839	MLZ	F
SMO	57272	Veracruz	La Puerta, 31 mi SW Orizaba by road	18.714682	-97.338439	MLZ	F
SMO	78507	Veracruz	Orizaba	18.8506857	-97.1002655	MCZ	F
SMO	OMVP52	Oaxaca	Puerto de La Soledad	18.16	-96.99	MZFC	F
SMO	OMVP1036	Oaxaca	San Martín Caballero	18.11	-96.64	MZFC	F
SMO	OMVP919	Oaxaca	Distrito de Cuicatlán	17.84	-96.75	MZFC	F
SMO	MT244	Oaxaca	Brecha 60, carr. Tuxtepec-Oaxaca	-96.513333	17.555	MZFC	F
SMO	OMVP598	Oaxaca	Sierra de Huautla, Puerto Soledad	-96.996667	18.165	MZFC	F
SMO	OMVP13	Oaxaca	Puerto de La Soledad	18.16	-96.99	MZFC	F
SMO	CONACYT777	Oaxaca	Puerto de La Soledad	18.16	-96.99	MZFC	F
SMO	CONACYT752	Oaxaca	Puerto de La Soledad	18.16	-96.99	MZFC	F
SMO	OR01	Oaxaca	Puerto de la Soledad, Teotitlán	-97.076664	18.13194556	MZFC	F
SMO	CONACYT756	Oaxaca	Puerto de La Soledad	18.16	-96.99	MZFC	F
SMO	37205	Oaxaca	Oaxaca	ND	ND	AMNH	F
SMO	815327	Oaxaca	Río Molino	ND	ND	AMNH	F
SMO	37226	Oaxaca	Oaxaca	ND	ND	AMNH	F
SMO	30935	Oaxaca	Moctum	17.247789	-96.004919	MLZ	F
GRO	163442	Guerrero	Chilpancingo	17.5507481	-99.5003136	MCZ	M
GRO	AMT133	Guerrero	Carrizal de Bravo	17.67	-99.88	MZFC	M
GRO	ATO117	Guerrero	Carrizal de Bravo	17.67	-99.88	MZFC	M

GRO	AMT049	Guerrero	Carrizal de Bravo	17.67	-99.88	MZFC	M
GRO	ATO120	Guerrero	Carrizal de Bravo	17.67	-99.88	MZFC	M
GRO	4696	Guerrero	Omiltemi, Laguna de Agua Fría	-100.066667	17.065	MZFC	M
GRO	MOLGRO164	Guerrero	Carrizal de Bravo	17.58	-99.83	MZFC	M
GRO	MOLGRO177	Guerrero	Carrizal de Bravo	17.58	-99.83	MZFC	M
GRO	MFOR512	Guerrero	ND	ND	ND	MZFC	M
GRO	482605	Guerrero	Chilpancingo	17.55	-99.5	AMNH	M
GRO	29702	Guerrero	Coapango	17.506199	-99.636357	MLZ	M
GRO	29703	Guerrero	Coapango	17.506199	-99.636357	MLZ	M
GRO	29699	Guerrero	Coapango	17.506199	-99.636357	MLZ	M
GRO	29697	Guerrero	Coapango	17.506199	-99.636357	MLZ	M
GRO	29701	Guerrero	Coapango	17.506199	-99.636357	MLZ	M
GRO	29698	Guerrero	Coapango	17.506199	-99.636357	MLZ	M
GRO	29696	Guerrero	Coapango	17.506199	-99.636357	MLZ	M
GRO	29700	Guerrero	San Vicente de Benitez	17.297901	-100.283612	MLZ	M
GRO	29695	Guerrero	San Vicente de Benitez	17.297901	-100.283612	MLZ	M
GRO	1312	Guerrero	Chilpancingo	17.550599	-99.505783	MLZ	M
GRO	1313	Guerrero	Chilpancingo	17.550599	-99.505783	MLZ	M
GRO	172633	Guerrero	Omiltemi	17.5507478	-99.666987	MCZ	M
GRO	172634	Guerrero	Omiltemi	17.5507478	-99.666987	MCZ	M
GRO	172635	Guerrero	Omiltemi	17.5507478	-99.666987	MCZ	M
GRO	172636	Guerrero	Omiltemi	17.5507478	-99.666987	MCZ	M
GRO	172639	Guerrero	Omiltemi	17.5507478	-99.666987	MCZ	M
GRO	172638	Guerrero	Omiltemi	17.5507478	-99.666987	MCZ	M
GRO	172640	Guerrero	Omiltemi	17.5507478	-99.666987	MCZ	M
GRO	172641	Guerrero	Omiltemi	17.5507478	-99.666987	MCZ	M
GRO	172642	Guerrero	Omiltemi	17.5507478	-99.666987	MCZ	M
GRO	172643	Guerrero	Omiltemi	17.5507478	-99.666987	MCZ	M

GRO	172644	Guerrero	Omiltemi	17.5507478	-99.666987	MCZ	M
GRO	172645	Guerrero	Omiltemi	17.5507478	-99.666987	MCZ	M
GRO	MOLGRO195	Guerrero	Carrizal de Bravo	17.58	-99.83	MZFC	F
GRO	MOLGRO231	Guerrero	Carrizal de Bravo	17.58	-99.83	MZFC	F
GRO	AGNS329	Guerrero	Puerto el Gallo, 15.5 km NNE Paraíso	-100.16667	17.46666667	MZFC	F
GRO	AMT155	Guerrero	Carrizal de Bravo	17.67	-99.88	MZFC	F
GRO	AGNS501	Guerrero	Omiltemi, Captación Potrerillos	-100.06667	17.065	MZFC	F
GRO	AGNS500	Guerrero	Omiltemi, Laguna de Agua Fría	-100.06667	17.065	MZFC	F
GRO	AGNS0192	Guerrero	Puerto el Gallo, 15.5 km NNE Paraíso	-100.16667	17.46666667	MZFC	F
GRO	3552	Guerrero	El Iris, 3 km NE Puerto El Gallo	-100.2	17.48333333	MZFC	F
GRO	482606	Guerrero	Chilpancingo	17.55	-99.5	AMNH	F
GRO	482607	Guerrero	Chilpancingo	17.55	-99.5	AMNH	F
GRO	29571	Guerrero	San Vicente de Benitez	17.297901	-100.283612	MLZ	F
GRO	1315	Guerrero	Chilpancingo	17.550599	-99.505783	MLZ	F
GRO	1317	Guerrero	Chilpancingo	17.550599	-99.505783	MLZ	F
GRO	1314	Guerrero	Chilpancingo	17.550599	-99.505783	MLZ	F
GRO	1316	Guerrero	Chilpancingo	17.550599	-99.505783	MLZ	F
GRO	172637	Guerrero	Omiltemi	17.5507478	-99.666987	MCZ	F
CHIS	EAGT809	Chiapas	Cerro Mozotal	15.42	-92.34	MZFC	M
CHIS	BMM877	Chiapas	Volcán Tacaná	15.06	-92.08	MZFC	M
CHIS	PEP376	Chiapas	Puerto del Gallo	-100.16667	17.46666667	MZFC	M
CHIS	MOL13052	Chiapas	Cerro Huitepec	16.73	-92.68	MZFC	M
CHIS	BMM875	Chiapas	Volcán Tacaná	15.06	-92.08	MZFC	M
CHIS	MOL13119	Chiapas	Cerro Huitepec	16.73	-92.68	MZFC	M
CHIS	EAGT813	Chiapas	Cerro Mozotal	15.42	-92.34	MZFC	M
CHIS	MOL13132	Chiapas	Cerro Huitepec	16.73	-92.68	MZFC	M
CHIS	44211	Chiapas	Tumbala	17.279187	-92.316808	MLZ	M
CHIS	44212	Chiapas	Tumbala	17.279187	-92.316808	MLZ	M
CHIS	36977	Chiapas	Volcan Tacana	15.123741	-92.119715	MLZ	M

CHIS	37484	Chiapas	Volcan Tacana	15.123741	-92.119715	MLZ	M
CHIS	37485	Chiapas	Volcan Tacana	15.123741	-92.119715	MLZ	M
CHIS	37486	Chiapas	Volcan Tacana	15.123741	-92.119715	MLZ	M
CHIS	37505	Chiapas	Volcan Tacana	15.123741	-92.119715	MLZ	M
CHIS	37493	Chiapas	Volcan Tacana	15.123741	-92.119715	MLZ	M
CHIS	36794	Chiapas	Volcan Tacana	15.123741	-92.119715	MLZ	M
CHIS	37483	Chiapas	Volcan Tacana	15.123741	-92.119715	MLZ	M
CHIS	45095	Chiapas	5 mi SW Ciudad Las Casas	16.706187	-92.689818	MLZ	M
CHIS	272900	Chiapas	Cerro Male, Porvenir	15.4508656	-92.2334889	MCZ	M
CHIS	272901	Chiapas	Cero Saxchanel, Sierra Madre	15.5050325	-92.6057268	MCZ	M
CHIS	394183	(Country of Guatemala)	Santa Ilania	ND	ND	AMNH	M
CHIS	74438	(Country of Guatemala)	Volcán de Fuego	ND	ND	MCZ	M
CHIS	74437	(Country of Guatemala)	Ridge above Calderas, Volcán de Fuego	ND	ND	MCZ	M
CHIS	113825	(Country of Guatemala)	Guatemala	ND	ND	MCZ	M
CHIS	145864	(Country of Guatemala)	Tecpam	ND	ND	MCZ	M
CHIS	145865	(Country of Guatemala)	Santa Ilaina	ND	ND	MCZ	M
CHIS	193620	(Country of Guatemala)	Zacapa, Usumatlán	ND	ND	MCZ	M
CHIS	MOL13297	Chiapas	Cerro Huitepec	16.73	-92.68	MZFC	F
CHIS	BONA90	Chiapas	Volcán Tacaná	15.06	-92.08	MZFC	F
CHIS	BMM861	Chiapas	Volcán Tacaná, Papales	-92.108333	15.13166667	MZFC	F
CHIS	MOL13076	Chiapas	Cerro Huitepec	16.73	-92.68	MZFC	F
CHIS	CRGA35	Chiapas	Cerro Boquerón	15.23	-92.3	MZFC	F

CHIS	EAGT828	Chiapas	Cerro Boquerón	15.23	-92.3	MZFC	F
CHIS	44225	Chiapas	Tumbala	17.279187	-92.316808	MLZ	F
CHIS	44224	Chiapas	Tumbala	17.279187	-92.316808	MLZ	F
CHIS	37491	Chiapas	Volcan Tacana	15.123741	-92.119715	MLZ	F
CHIS	37511	Chiapas	Volcan Tacana	15.123741	-92.119715	MLZ	F
CHIS	37496	Chiapas	Volcan Tacana	15.123741	-92.119715	MLZ	F
CHIS	272899	Chiapas	Cero Male	15.4508656	-92.2334889	MCZ	F
CHIS	272902	Chiapas	Cero Saxchanel, Sierra Madre	15.5050325	-92.6057268	MCZ	F
CHIS	272903	Chiapas	Rodeo, Siltepec	15.4758643	-92.2718298	MCZ	F
CHIS	193619	(Country of Guatemala)	Usumatlan, 8 mi NW	ND	ND	MCZ	F
CA	18_197	Chalatenango	Los Esesmiles	ND	ND	BMC	M
CA	18_198	Chalatenango	Los Esesmiles	ND	ND	BMC	M
CA	18_ 200	Chalatenango	Los Esesmiles	ND	ND	BMC	M
CA	18_233	Chalatenango	Los Esesmiles	ND	ND	BMC	M
CA	18_236	Chalatenango	Los Esesmiles	ND	ND	BMC	M
CA	18_489	Chalatenango	Los Esesmiles	ND	ND	BMC	M
CA	18_547	Chalatenango	Los Esesmiles	ND	ND	BMC	M
CA	18_563	Chalatenango	Los Esesmiles	ND	ND	BMC	M
CA	18302	Ocotepeque	Monte Verde	14.5166664	-88.7499962	MLZ	M
CA	18305	Ocotepeque	Monte Verde	14.5166664	-88.7499962	MLZ	M
CA	18306	Ocotepeque	Monte Verde	14.5166664	-88.7499962	MLZ	M
CA	18308	Ocotepeque	Monte Verde	14.5166664	-88.7499962	MLZ	M
CA	18301	Ocotepeque	Monte Verde	14.5166664	-88.7499962	MLZ	M
CA	18303	Ocotepeque	Monte Verde	14.5166664	-88.7499962	MLZ	M
CA	18307	Ocotepeque	Monte Verde	14.5166664	-88.7499962	MLZ	M
CA	18304	Ocotepeque	Monte Verde	14.5166664	-88.7499962	MLZ	M
CA	18309	Ocotepeque	Monte Verde	14.5166664	-88.7499962	MLZ	M
CA	18310	Ocotepeque	Monte Verde	14.5166664	-88.7499962	MLZ	M

CA	25499	La Paz	El Caliche	ND	ND	MLZ	M
CA	16835	Ocotepeque	Montaña El Sillón	14.4010972	-89.0833282	MLZ	M
CA	16836	Ocotepeque	Montaña El Sillón	14.4010972	-89.0833282	MLZ	M
CA	17009	Ocotepeque	Montaña El Sillón	14.4010972	-89.0833282	MLZ	M
CA	17007	La Paz	Montana La Cruz	ND	ND	MLZ	M
CA	17008	La Paz	Montana La Cruz	ND	ND	MLZ	M
CA	15584	La Paz	Montana La Cruz	ND	ND	MLZ	M
CA	16837	Ocotepeque	Montaña El Chorro	14.4333334	-89.0833282	MLZ	M
CA	16834	Ocotepeque	Montaña El Chorro	14.4333334	-89.0833282	MLZ	M
CA	15454	Ocotepeque	Monte El Candado	14.4333334	-89.0499992	MLZ	M
CA	17099	Ocotepeque	Montaña El Portillo	14.4666672	-89.0666695	MLZ	M
CA	161254	(Country of Honduras)	Montaña Vázquez	ND	ND	MCZ	M
CA	161256	(Country of Honduras)	Montaña Vázquez	ND	ND	MCZ	M
CA	161257	(Country of Honduras)	Montaña Vázquez	ND	ND	MCZ	M
CA	161258	(Country of Honduras)	Montaña Vázquez	ND	ND	MCZ	M
CA	161259	(Country of Honduras)	Montaña Vázquez	ND	ND	MCZ	M
CA	161260	(Country of Honduras)	Montaña Vázquez	ND	ND	MCZ	M
CA	161261	(Country of Honduras)	Montaña Vázquez	ND	ND	MCZ	M
CA	161262	(Country of Honduras)	Montaña Vázquez	ND	ND	MCZ	M
CA	161263	(Country of Honduras)	Montaña Vázquez	ND	ND	MCZ	M

CA	161265	(Country of Honduras)	Cerro Cantoral	ND	ND	MCZ	M
CA	161268	(Country of Honduras)	Cerro Cantoral	ND	ND	MCZ	M
CA	161270	(Country of Honduras)	Cerro Cantoral	ND	ND	MCZ	M
CA	169855	(Country of Honduras)	Cantoral	ND	ND	MCZ	M
CA	161264	(Country of Honduras)	Montaña Vázquez	ND	ND	MCZ	M
CA	161267	(Country of Honduras)	Montaña Vázquez	ND	ND	MCZ	M
CA	161269	(Country of Honduras)	Montaña Vázquez	ND	ND	MCZ	M
CA	158121	(Country of Honduras)	Montaña Vázquez	ND	ND	MCZ	M
CA	265213	(Country of Honduras)	Montaña Vázquez	ND	ND	MCZ	M
CA	18_194	Chalatenango	Los Esesmiles	ND	ND	BMC	F
CA	18_467	Chalatenango	Los Esesmiles, Chalatenango	ND	ND	BMC	F
CA	18_468	Chalatenango	Los Esesmiles, Chalatenango	ND	ND	BMC	F
CA	18_487	Chalatenango	Los Esesmiles, Chalatenango	ND	ND	BMC	F
CA	18313	Ocotepeque	Monte Verde	14.5166664	-88.7499962	MLZ	F
CA	18312	Ocotepeque	Monte Verde	14.5166664	-88.7499962	MLZ	F
CA	18311	Ocotepeque	Monte Verde	14.5166664	-88.7499962	MLZ	F
CA	17098	Ocotepeque	Montaña El Portillo	14.4666672	-89.0666695	MLZ	F
CA	17097	Ocotepeque	Montaña El Portillo	14.4666672	-89.0666695	MLZ	F
CA	17011	La Paz	Montana La Cruz	ND	ND	MLZ	F
CA	16664	La Paz	Montana La Cruz	ND	ND	MLZ	F

CA	16842	La Paz	Montana La Cruz	ND	ND	MLZ	F
CA	16667	La Paz	Montana La Cruz	ND	ND	MLZ	F
CA	16668	La Paz	Montana La Cruz	ND	ND	MLZ	F
CA	16666	Ocotepeque	Montaña El Chorro	14.4333334	-89.0833282	MLZ	F
CA	169853	(Country of Honduras)	Cerro Cantoral	ND	ND	MCZ	F
CA	169854	(Country of Honduras)	Cerro Cantoral	ND	ND	MCZ	F

ND: No data.
BC: Biological Collection.
AMNH: The American Museum of Natural History.
BMC: The Donald R. Dickey Bird and Mammal Collection, University of California Los Angeles.
MCZ: The Museum of Comparative Zoology, Harvard University.
MLZ: The Moore Lab of Zoology, Occidental College.
MZFC: Museo de Zoología, Facultad de Ciencias (Alfonso L. Herrera), Universidad Nacional Autónoma de México.
