# Supplementary material for: Genetic and morphometric divergence in the Garnet-Throated Hummingbird Lamprolaima rhami (Aves: Trochilidae)
Source: PeerJ. 2018 Oct 19;6:e5733. doi: 10.7717/peerj.5733 (PMC6197039; doi:10.7717/peerj.5733)
Supplement: Table S3 — Primers and PCR protocols used in this study. Arévalo E, Davis SK, Sites JW. 1994. Mitochondrial DNA sequence divergence and phylogenetic relationships among eight chromosome races of the Sceloporus grammicus complex (Phrynosomatidae) in central Mexico. Systematic Biology 43:387–418 DOI 10.1093/sysbio/43.3.387. Eberhard JR, Bermingham E, Zink R. 2004. Phylogeny and biogeography of the Amazona ochrocephala (Aves: Psittacidae) complex. The Auk 121:318–332 DOI 10.1642/0004-8038(2004)121[0318:PABOTA]2.0.CO;2. Prychitko TM, Moore WS. 1997. The utility of DNA sequences of an intron from the β-fibrinogen gene in phylogenetic analysis of woodpeckers (Aves: Picidae). Molecular Phylogenetics and Evolution 8:193–204 DOI 10.1006/mpev.1997.0420. Sorenson MD, Ast JC, Dimcheff DE, Yuri T, Mindell DP. 1999. Primers for a PCR-based approach to mitochondrial genome sequencing in birds and other vertebrates. Molecular Phylogenetics and Evolution 12:105–114 DOI 10.1006/mpev.1998.0602. [file peerj-06-5733-s003.pdf]

Supplemental Information S3

Primers and PCR protocols used in this study.

Gene	Primer name	Primer sequence	References	PCR protocol		
				Denaturation	Annealing (35X)	Extension
ND2	L5219	CCCATACCCCGAAAATGATG	Sorenson *et al.*, 1999.	1x 03:00(94°C)	00:30(94°C), 00:30(54°C), 00:45(72°C)	1x 10:00(72°C)
ND2	H6313	CTCTTATTTAAGGCTTTGAAGGC				
ND4	ND4	CACCTATGACTACCAAAAGCTCATGTAGAAGC	Arévalo *et al.*, 1994.	1x 05:00(94°C)	00:30(95°C), 00:30(55°C), 00:45(72°C)	1x 07:00(72°C)
ND4	LEU	CATTACTTTTACTTGGATTTGCACCA				
ATPase6 and 8	CO2GQL	GGACAATGCTCAGAAATCTGCGG	Eberhard et al., 2004	1x 03:00(94°C)	00:30(94°C), 00:30(58°C), 00:45(72°C)	1x 10:00(72°C)
ATPase6 and 8	CO3HMH	CATGGGCTGGGGTCRACTATGTG				
CR	ARCOIF	AATTTTATGGTCTTTGTGTGTGAA	González et al. 2011	1x 03:00(94°C)	00:30(94°C), 00:30(50°C), 00:45(72°C)	1x 10:00(72°C)
CR	ARCOIR	ACCCTAGCACAACTCGCACT				
BFib	BFib-17L2	TGGGAGGTGAAGCAGCTAAGAAAAACAA	Prychitko & Moore, 1997.	1x 10:00(94°C)	01:00(92°C), 01:00(50°C), 01:00(72°C)	1x 07:00(72°C)
BFib	BFib-17U2	CATCCATGCAGTTCTGGCAATTC				
MUSK	MUSK-F3	GCTGTACTTCCATGCACTACAATG	McGuire *et al.*, 2014.	1x 05:00(95°C)	00:25(95°C), 00:25(50°C), 01:00(72°C)	1x 07:00(72°C)
MUSK	MUSK-R3	ATCCTCAAATTTCCCGAATCAAG				
ODC	ODC-2F	GCGTGCAAAAGAACTTGACC	McGuire *et al.*, 2014.	1x 03:00(94°C)	00:30(94°C), 00:30(57°C), 00:30(72°C)	1x 05:00(72°C)
ODC	ODC-2R	AGCCACCACCAATATCAAGC				
