# Supplementary material for: Genetic and morphometric divergence in the Garnet-Throated Hummingbird Lamprolaima rhami (Aves: Trochilidae)
Source: PeerJ. 2018 Oct 19;6:e5733. doi: 10.7717/peerj.5733 (PMC6197039; doi:10.7717/peerj.5733)
Supplement: Table S4 — Lilliefors (Kolmogorov-Smirnov) results to test normality of the morphometric data. Test of Wilcoxon/Mann-Whitney to evaluate differences between males and females. Kruskall–Wallis tests to evaluate significant differences between paired geographic groups (SMO, GRO, CHIS, CA). Below diagonal: males, above diagonal: females. ∗p- value < 0.05, ∗∗p- value < 0.01. ∗∗∗p- value < 0.001. [file peerj-06-5733-s004.pdf]

Supplemantal information S4

Lilliefors (Kolmogorov-Smirnov) results to test normality of the morphometric data.

		UNCORRECTED DATA		CORRECTED DATA (log-transformation)	
		D	*p*-value	D	*p*-value
MALES	Bill length	0.054972	0.4123	0.057592	0.3394
	Bill width	0.06995	0.09483	0.096836	**0.002935**
	Bill depth	0.1015	**0.001389**	0.082337	**0.02284**
	Wing chord	0.091529	**0.006226**	0.10299	**0.001019**
	Tail length	0.092575	**0.00617**	0.1023	**0.001372**
FEMALES	Bill length	0.081525	0.3631	0.074573	0.5069
	Bill width	0.068054	0.6071	0.075687	0.4343
	Bill depth	0.087028	0.2568	0.067232	0.6603
	Wing chord	0.23253	**7.92E-10**	0.21699	**1.72E-08**
	Tail length	0.16319	**0.0001559**	0.15024	**0.0008004**

Test of Wilcoxon/Mann-Whitney to evaluate differences between males and females.

	W	*p*-value
Bill length	6035	**3.63E-06**
Bill width	5843	**0.004239**
Bill depth	7172	**5.97E-12**
Wing chord	8327	**2.20E-16**
Tail length	7944	**2.20E-16**

Kruskall-Wallis tests to evaluate significant differences between paired geographic groups (SMO, GRO, CHIS, CA). *Below diagonal*: males, *above diagonal*: females. **p*-value<0.05, ***p*-value<0.01. ****p*-value<0.001.

Bill length	SMO	GRO	CHIS	CA
SMO	---	3.5738	0.47915	2.633
GRO	3.6541	---	**7.8077****	0.28482
CHIS	0.78686	1.1404	---	**4.411***
CA	0.03264	**7.3222****	2.0731	---

Bill width	SMO	GRO	CHIS	CA
SMO	---	0.13414	0.20259	**9.953****
GRO	1.9535	---	0.18922	**7.6985****
CHIS	0.55619	0.82187	---	**7.7066****

CA	**15.34*** **	**4.6982***	**12.269*** **	---

Bill depth	SMO	GRO	CHIS	CA
SMO	---	**6.0137***	1.6954	**14.834*** **
GRO	**4.9483***	---	1.9715	**6.0147***
CHIS	0.12252	**9.4637** **	---	**9.6839** **
CA	**13.908*** **	0.75679	**22.012*** **	---

Wing chord	SMO	GRO	CHIS	CA
SMO	---	**12.942*** **	1.37227	**7.1119** **
GRO	0.3494	---	**15.785*** **	**21.26*** **
CHIS	0.055364	0.060538	---	1.5062
CA	**15.655*** **	**7.6503** **	**11.681*** **	---

Tail length	SMO	GRO	CHIS	CA
SMO	---	**9.2154** **	0.86099	**9.6551** **
GRO	3.4614	---	**6.5611***	**19.203*** **
CHIS	**5.3036***	0.20569	---	**11.884*** **
CA	3.13	**7.4467** **	**14.044*** **	---
